# Biochar to improve the thermal performance of living wall systems: laboratory assessment of three planting substrates

**DOI:** 10.1007/s42773-025-00508-5

**Published:** 2026-01-10

**Authors:** Josh Batterham, João Alencastro, Thomas Murphy, Jack Morewood, Steve Goodhew

**Affiliations:** 1 School of Art, Design and Architecture, University of Plymouth, Plymouth, UK; 2 School of Geography, Earth and Environmental Sciences, University of Plymouth, Plymouth, UK

**Keywords:** Living wall systems, Nature-based solutions, Building energy performance, Moisture, Thermal conductivity, Green infrastructure

## Abstract

Living wall systems (LWSs) help to alleviate the climate and biodiversity harms associated with buildings and bring benefits to building occupants. Their performance can be variable and existing research points to the planting substrate as a key design factor. This study provides quantitative evidence on the physical, thermal and moisture performance of three planting substrates that vary according to the proportion of biochar added to green waste compost (GWC). Thermal conductivity (Wm^−1^ K^−1^), thermal resistivity (mK W^−1^), volumetric moisture content (%) and mass (g) are measured for each fraction, replicated six times. Controlled drying procedures were employed, measuring these properties at a range of moisture levels. Data analysis finds that volumetric moisture content and biochar fraction have a statistically significant (*p* ≤ 0.05) effect on thermal conductivity. Added biochar is associated with non-linear reductions in thermal conductivity at low moisture levels. This suggests increasing the biochar fraction while reducing moisture in the substrate of a LWS will reduce its thermal conductivity, with a 100 mm planting substrate with 30% biochar and 30%vol moisture content providing 0.82 m^2^ KW^−1^ of thermal resistance, compared to 0.46 m^2^ KW^−1^ without added biochar. The methods build on previous work to assess the properties of different planting substrates for LWSs, providing a practical, lab-based assessment of biochar. The data produced are useful for researchers and professionals seeking to understand how biochar additions impact irrigation and thermal performance when specifying and designing LWSs and underline the potential value of biochar for improving the thermal performance of green infrastructure more widely.

**Graphical Abstract:**

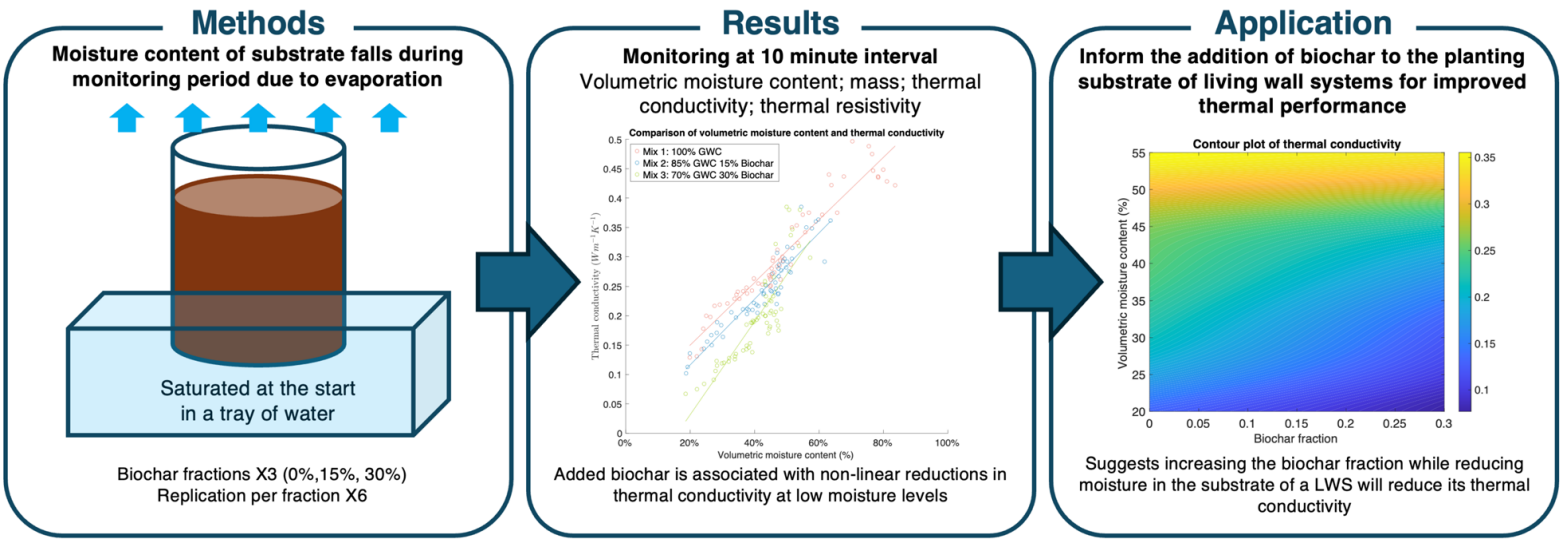

## Highlights

- LWSs bring a range of benefits, but evidence is needed to ensure appropriate planting substrate and irrigation.
- Laboratory assessment of thermal performance when adding biochar to green waste compost for a range of moisture levels.
- Non-linear thermal conductivity reductions at low moisture with added biochar.

## Introduction

The built environment is responsible for 34% of energy demand and 37% of carbon emissions globally (Global Alliance for Buildings and Construction 2024). The climate crisis is intertwined with many other crises, least of all the global loss of biodiversity (WWF 2022; Ripple et al. 2024). Nature-based solutions such as LWSs can help to mitigate both the climate and biodiversity harms attributed to buildings (UK Green Building Council 2022; CIOB 2023, pp. 179–186) and have been found to provide extensive benefits for the performance of buildings (Manso et al. 2021).

LWSs are a specific type of vertical greening system that involve installing a substrate on a wall into which plants root and grow (Bustami et al. 2018). LWSs include modular (Manso and Castro-Gomes 2015; Bustami et al. 2018), linear (Medl et al. 2017), continuous (Manso and Castro-Gomes 2015; Oquendo-Di Cosola et al. 2022) and double plant-skin façade systems (Bao et al. 2023). LWSs require irrigation and nutrition which are often provided by a watering system (Bustami et al. 2018). As the adoption of LWSs grows, this paper provides a practical, lab-based way to assess different planting substrates for use in LWSs at the design stage of a LWS project.

### The benefits attributed to living wall systems

LWSs contribute to improved indoor and outdoor environmental quality, as plants in these systems purify the air through botanical biofiltration, which requires less energy and lower maintenance than a mechanical ventilation system (Alvarado-Alvarado et al. 2024). In-situ monitoring has found that LWSs contribute to reductions in carbon dioxide (CO2) concentration (Dominici et al. 2021), volatile organic compounds (VOCs) (Alvarado-Alvarado et al. 2024) and particulate matter (PM) (Shao et al. 2021; Koch et al. 2023). This depends on the planting substrate (Alvarado-Alvarado et al. 2024), planting (Hellebaut et al. 2022; Koch et al. 2023; Falzone et al. 2024), ventilation (Plitsiri and Taemthong 2024), size of the LWS (Mobarhan et al. 2024) and orientation (Qian et al. 2024).

LWSs have a cooling effect (Shafiee et al. 2020; Daemei et al. 2021; Ruiz-Valro et al. 2022; Bakhtyari et al. 2024) with some studies monitoring external surface temperature reductions of up to 14 °C (Cortês et al. 2022). This helps to reduce the urban heat island phenomenon outdoors and space cooling demand indoors. Cooling is greater on the walls most exposed to solar radiation (Cortês et al. 2022). LWSs provide cooling in many different ways: shading walls from solar radiation by plants, by evapotranspiration from plants, reducing airflow over the surface of walls, absorbing solar radiation and altering diffuse reflection of sunlight (Cameron et al. 2014).

LWSs provide additional insulation (Abdeen and Rafaat 2024). Steady-state modelling for a variety of wall types by Vijayalaxmi and Gandham (2023) found that LWSs increase thermal resistance by between 12.76% for an insulated cavity wall and 93.60% for an uninsulated brick wall. In a comparative monitoring study in the United Kingdom, Fox et al. (2022) found that a solid masonry wall with an external LWS applied had an in-situ U-value of 0.77 Wm^−2^ K^−1^, compared to 1.12 Wm^−2^ K^−1^ without an external LWS, reducing thermal transmittance by 31%. Tudiwer and Korjenic (2017) carried out a similar experiment with a green façade and LWS in Vienna, finding that thermal resistance increased by 0.31 m^2^ KW^−1^ for a modular LWS and 0.68 m^2^ KW^−1^ for a continuous LWS. Nan et al. (2020) isolated the impact of plants and substrate by comparing a bare and planted wall, attributing insulation benefits to each component. The extent of the insulating effect depends on the existing wall construction, as well as plant-and building-specific factors (Karimi et al. 2022).

### How planting substrates affect the performance of LWSs

The performance of LWSs is variable (Manso et al. 2021) and achieving their benefits depends on many design factors. These include orientation (Nan et al. 2020; Bakhtyari et al. 2024), the typology (Bakhshoodeh et al. 2022), the mounting system (Rowe et al. 2022; Jimenez et al. 2024), the properties of the wall the LWS is attached to (Vijayalaxmi and Gandham 2023), any gap between the wall and LWS (Bakhshoodeh et al. 2022), access to light (Dominici et al. 2021), plant selection (Nan et al. 2020; Cortês et al. 2021; Koch et al. 2023), irrigation (Kaltsidi et al. 2020) and substrate (Dede et al. 2021).

The substrate provides added thermal resistance, but this depends on its conductivity (Nan et al. 2020; Vijayalaxmi and Gandham 2023). Higher moisture content is associated with increased thermal conductivity (Usowicz et al. 2016) alongside particle size, organic matter and compaction (Lunt et al. 2023). Fox et al. (2022) suggest that low-density soils with a greater porosity and organic matter may provide greater thermal insulation, although empirical data are needed to support this. Moisture content can only be reduced to an extent because the substrate must also support the plants which root into it by enabling both adequate nutrition (Dede et al. 2021) and irrigation (Kaltsidi et al. 2020).

So far, research into the performance of LWSs suggests that thermal conductivity varies between different substrates and is not comparable to modern insulation materials (Table 1). Usowicz et al. (2016) measured the thermal properties of pure biochar, finding that a mix of fraction sizes led to an increase in thermal conductivity. This was explained by higher bulk density, which indicates that smaller particles filled gaps between larger ones and thus reduced porosity. Libessart and Kenai (2018) studied five different substrates, finding that a coconut substrate achieved the lowest thermal conductivity. Lunt et al. (2023) found that a biochar-coconut substrate had the lowest thermal conductivity of three substrates across a range of moisture contents. Biochar-coconut was also able to hold water, maintain the same density and contain pockets with large volumes of air. This agrees with wider findings that organic matter content decreases thermal conductivity as it is associated with increased porosity (Zhu et al. 2019).

**Table 1:** Existing research into the thermal conductivity of different LWS planting substrates

Ref	Substrate	Composition	Method	State	Thermal conductivity (Wm^−1^ K^−1^)
Usowicz et al. (2016)	Biochar	100% biochar with increasing fraction sizes	Thermal conductivity was measured in laboratory conditions with a range of textured fractions measured by their diameter	Diameter under 0.5 mm	0.079
				Diameter 0.5–1 mm	0.08
				Diameter 1–2 mm	0.078
				Diameter 2–5 mm	0.08
				Diameter over 5 mm	0.104
				Mix of all above fractions	0.132
Libessart and Kenai (2018)	Sand	100% sand	Thermal conductivity was measured in laboratory conditions after being allowed to dry for 48 h in a steam room	Dry	0.225
	Universal plant mix	Composted bark, fair peat and plant compost		Dry	0.062
	Clay balls	100% natural clay		Dry	0.105
	Sphagnum	100% Sphagnum moss		Dry	0.06
	Manufacturer substrate	90% coconut matting, 10% perlite		Dry	0.051
Lunt et al. (2023)	FabSoil	China clay mining waste and bark material	Thermal conductivity was measured in laboratory conditions for a range of moisture content in the compacted and uncompacted state	Dry compacted	0.2–0.9
				Dry uncompacted	0.2–0.6
	Green-waste compost	100% green-waste compost		Dry compacted	0.2–0.8
				Dry uncompacted	0.2–0.8
	Biochar-coconut	70% coconut coir, 20% biochar, 10% seaweed mixture		Dry compacted	0.2–0.5
				Dry uncompacted	0.2–0.5

### The potential role of biochar in improving the performance of LWSs

Biochar is created when biomass is decomposed under oxygen-limited conditions (pyrolysis) and has been in use since the Palaeolithic era when wood was decomposed into charcoal (Chen et al. 2019). Biochar is receiving new attention among researchers (Wu et al. 2024) thanks to its status as an ‘inertinite’, being stable at geological timescales (Sanei et al. 2024), and multiple applications in supporting carbon neutrality (Wang et al. 2023). Biochar can also act as a sustainable alternative to inorganic fertilisers due to its better nutrient retention and mitigation of greenhouse gas emissions (Mohammadi et al. 2025). The addition of biochar to soils influences both the water-holding capacity and hydrophobicity (Adhikari et al. 2022), and promotes microbial colonisation (Bolan et al. 2023).

There is limited research into the impact of biochar on LWSs specifically; existing studies find that biochar additions are associated with moisture retention of LWS (Kraus et al. 2020) and may improve treatment of greywater in LWS (Sami et al. 2023). However, there are no previous studies assessing biochar application for thermal performance of LWSs. More literature exists on biochar additions to green roofs, with studies finding that additions increase water retention (Petreje et al. 2025), reduce surface runoff (Petreje et al. 2025) and reduce substrate erosion (Liao et al. 2022). Biochar has been found to increase porosity and reduce the bulk density when added to soils (Dayoub et al. 2023; Acharya et al. 2024), suggesting it may be suitable to improve insulation and reduce the weight-bearing requirements of LWSs.

Biochar additions could also improve plant growth. Biochar has been found to positively influence seed germination and seedling growth due to its effects on water-holding capacity of substrates and available minerals (Ali et al. 2021). The alkaline nature of biochar has also been found to increase pH in some soils (Dayoub et al. 2023) which could improve plant growth. A meta-analysis by Xiang et al. (2017) found that biochar additions improved root traits, increasing root biomass, volume, surface area, length and the number of root tips. Biochar may also support plant performance through associated changes in microbial diversity and metabolic potential in the rhizosphere (Kolton et al. 2017). High ratios of biochar could, however, negatively affect plant performance (Gale and Thomas 2019). The benefits of biochar additions for plant growth depend on dose, plant species (Gale and Thomas 2019) and biochar feedstock (Gascó et al. 2016; Xu et al. 2024).

However, there are however aspects of biochar that have not yet been explored fully, such as its effects on substrates over a long period of time of ten to twenty years (Spokas 2010) and how moderation of moisture environment regulates the soil environment for long-term plant performance.

### Research questions

Much of the existing work on the design factors of LWSs has tended to focus away from the substrate and around plant selection or mounting system. Yet recent work has positioned the substrate as a key design factor in achieving the benefits of LWSs, affecting biofiltration (Alvarado-Alvarado et al. 2024), sound absorption (Oquendo-Di Cosola et al. 2022), water management (Aicher et al. 2022; Anangadan et al. 2024) and thermal performance (Nan et al. 2020; Fox et al. 2022; Vijayalaxmi and Gandham 2023). At the same time, there is a need to assess alternative substrates such as biochar which could bring benefits to LWSs because of its increased porosity, reduced bulk density and role in improving plant growth.

Because of the “lack of empirical data on the effects of living wall planting substrate on building insulation” (Fox et al. 2022), the substrate became the focus of this study. Evidence on the physical, thermal and moisture properties of different LWS planting substrates is needed to make informed decisions about whether biochar-enhanced substrates work well alongside other design parameters and climatic conditions (Dede et al. 2021).

This paper therefore assesses the impact of added biochar on the thermal and moisture performance of LWS planting substrate using a practical, lab-based test. The study had two principal research questions: The research questions (RQs) were:

- RQ1: Do additions of biochar reduce the thermal conductivity of LWS planting substrate and improve the thermal performance of a LWS?
- RQ2: How do biochar additions influence moisture retention and LWS irrigation?

Answering these questions will help to address variability in performance (Manso et al. 2021) and empower professionals with data to optimise performance when planning and designing LWSs.

## Materials and methods

### Description of the three planting substrates

**Fig. 1:**
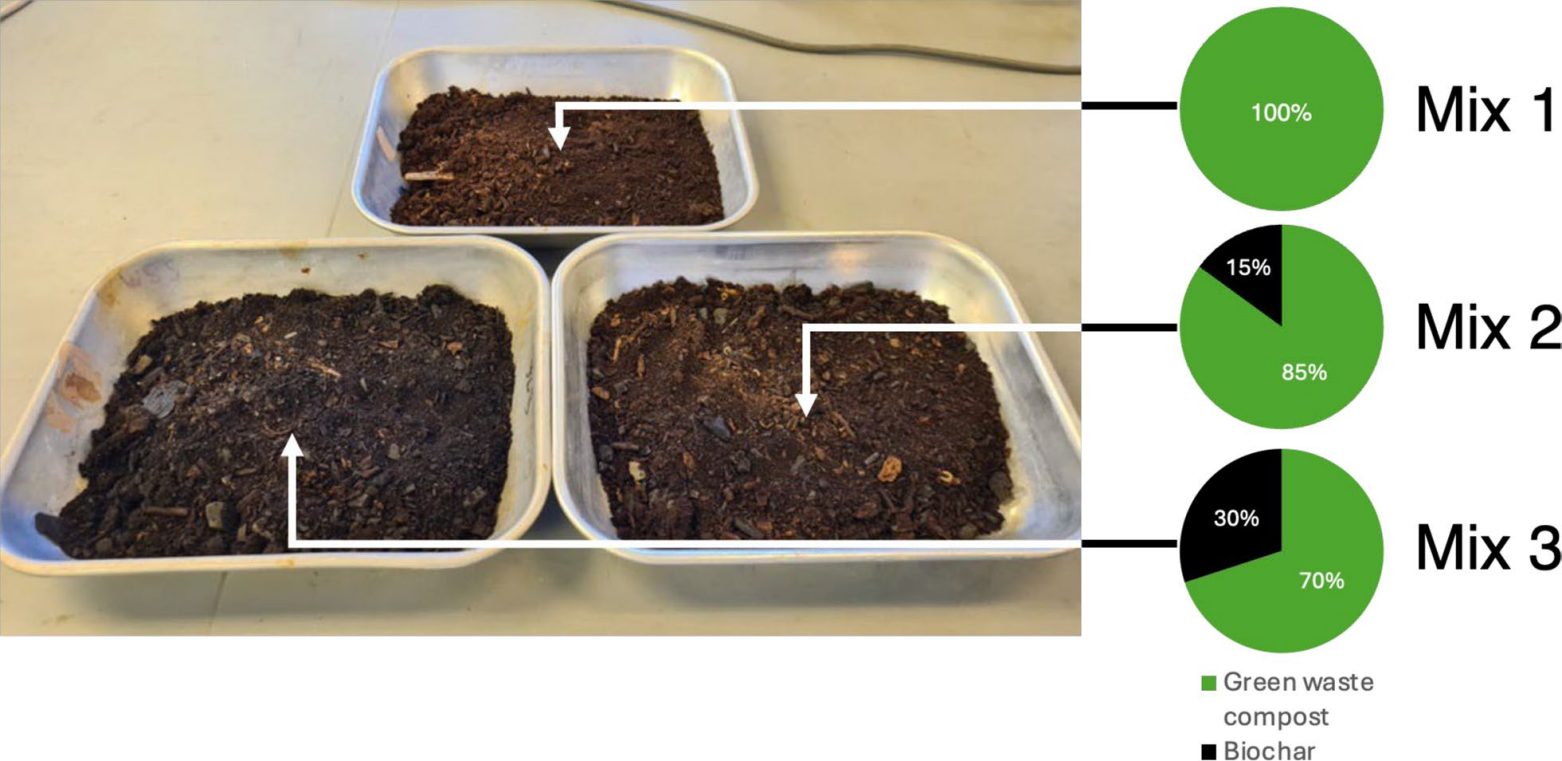
The composition of the three planting substrates. Photo: Josh Batterham

Three planting substrates were compared in this investigation which are characterised by different ratios of GWC and biochar (Fig. 1). Two had added biochar and one used only GWC to provide a baseline.

The biochar was manufactured by Trimplants Wholesale Nursery (Trimplants 2024), Combe Raleigh, Devon, UK using local hardwood in Dartmoor Dragon biochar retorts (Charcoal 2024). This biochar is characterized by a compact retort and smaller feedstock, such as hedge laying arising and smaller diameter branches. Pyrolysis takes place in a metal barrel which creates a controlled, oxygen-free pyrolysis environment at 650–800 °C. Based on data provided by the manufacturer, the surface area ranges from 300 to 1000 m^2^ g^–1^, depending on feedstock and processing conditions. The biochar has a high microporosity, is aklaline (pH ~ 9–11) and high C/O ratio indicating increased stability and a carbon-rich structure. The high temperature is associated with higher electrical conductivity, higher nutrient absorption and lower volatile matter (Table 2)

**Table 2:** Physico-chemical properties of the biochar used based on data from the manufacturer Trimplants

Property	Value
Surface area	300–1000 m^2^g^–1^
pH	~ 9–11
C/O ratio	High
Feedstock	Ash tree

Samples were measured into one litre volumes and were well mixed using a barrel mixer for one minute each. This involved 30 turns of the mixer to improve the homogeneity of the material. Substrates were sourced locally and divided into 100 mm wide columns which represent a standardised protocol for substrate testing (Lunt et al. 2023). Because they are non-homogenous materials, and some variation can be expected in the planting substrate, six samples of each mix were created.

### Measurement

The 18 samples were then placed in a 3-in-deep tray of water for 24 h to fully saturate them after which any excess water was then poured away. The samples were placed in a 40 °C oven for 10 min and then allowed to return to room temperature over a further 10 min.

Appropriate apparatus with sufficient accuracy and range were specified (Table 3). Thermal conductivity and thermal resistivity were measured using a Decagon KD2 Pro with a single needle TR-1 sensor (10 cm long, 2.5 mm diameter), which uses the transient line heat source method (Decagon Devices Inc. 2016). Volumetric moisture content was measured using a Delta-T Thetaprobe at each stage (Delta-T Devices Ltd. 2017). All apparatus was calibrated in accordance with recommended manufacturer practices.

**Table 3:** Summary of measurement apparatus

Measurand	Unit	Apparatus used	Accuracy	Range
Thermal conductivity	Wm^−1^ K^−1^	Decagon KD2 Pro with TR-1	± 10% from 0.2 to 4.0	0.1–4.0
Thermal resistivity	mKW^−1^	Decagon KD2 Pro with TR-1	–	25–1000
Volumetric moisture content	%	Delta-T Thetaprobe	± 1 from 0 to 100	0–100

Measurements were taken before and after saturation, and then at 10-min intervals. This allowed the substrates to be dynamically monitored as moisture content fell due to evaporation. Monitoring continued until the samples fell below an average of 20% volumetric moisture, which was achieved after 580 min for all mixes. Further monitoring below this level would not be useful as plants consume water (Pradhan et al. 2019) and require moisture in the substrate to produce healthy vegetation. Once the experiment was completed, particle size analysis was conducted which involved the different samples being sieved within a range of 16 mm to > 2 mm.

### Data analysis and modelling

Statistical analysis was conducted in MATLAB, which included bivariate and multivariate linear regression modelling to understand the interactions between different variables and thermal conductivity. Statistical significance was determined using a significance level (α) of 0.05. This was compared to a Gaussian Process Regression model to understand whether a non-linear model could better explain the relationships between variables.

## Results and analysis

### Planting substrate measurement results

The physical properties of the three planting substrates were analysed in both their saturated and unsaturated states (Table 4). In their starting states, physical properties were similar. Once saturated, however, the volumetric moisture content of mix 1 was 19.33% vol higher than that of mix 3 with 30% added biochar. This suggests that the addition of biochar lowers the maximum saturation. The added biochar was associated with reduced mass when saturated, with mix 1 being 73.11 g heavier than mix 3. Meanwhile, thermal conductivity was lowest in biochar mixes 2 and 3. Although mix 2 contained a proportion of biochar between mixes 1 and 3, thermal conductivity at total saturation was the lowest of all three mixes at 0.25 Wm^−1^ K^−1^.

**Table 4:** Initial planting substrate data

	Mix 1: 100% GWC	Mix 2: 85% GWC 15% biochar	Mix 3: 70% GWC 30% biochar
Starting mass (g) (± standard deviation)	665.60 (± 14.03)	650.40 (± 2.88)	656.42 (± 28.56)
Starting volumetric moisture content (%vol)	15.77% (± 2.62%)	14.30% (± 1.79%)	16.70% (± 2.90%)
Wet mass (g)	1044.73 (± 26.09)	989.25 (± 37.39)	971.62 (± 22.95)
Saturated volumetric moisture content	85.92% (± 1.01%)	79.80% (± 2.87%)	66.58% (± 5.13%)
Saturated thermal conductivity (Wm^−1^ K^−1^)	0.48 (± 0.18)	0.25 (± 0.08)	0.32 (± 0.70)
Saturated thermal resistivity (m KW^−1^)	2.28 (± 1.05)	4.32 (± 1.28)	3.26 (± 0.74)

**Fig. 2:**
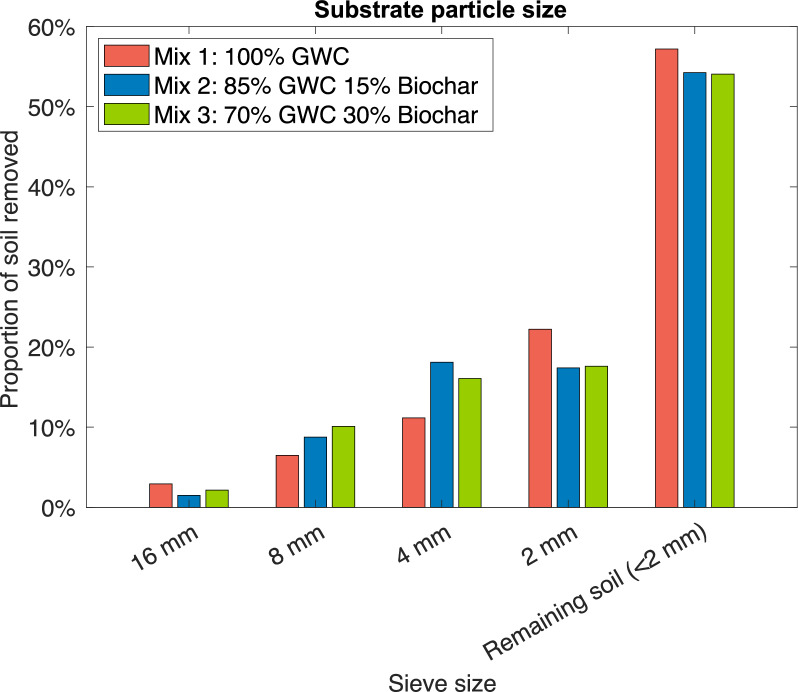
Planting substrate particle size

Particle size sieve analysis showed that particle sizes were similar amongst all mixes. Mix 1 had a marginally higher proportion of smaller particles (< 4 mm), whereas the other mixes had a marginally higher proportion of particles sized between 4 and 16 mm (Fig. 2), likely reflecting the biochar material added.

### Planting substrate monitoring results

**Fig. 3:**
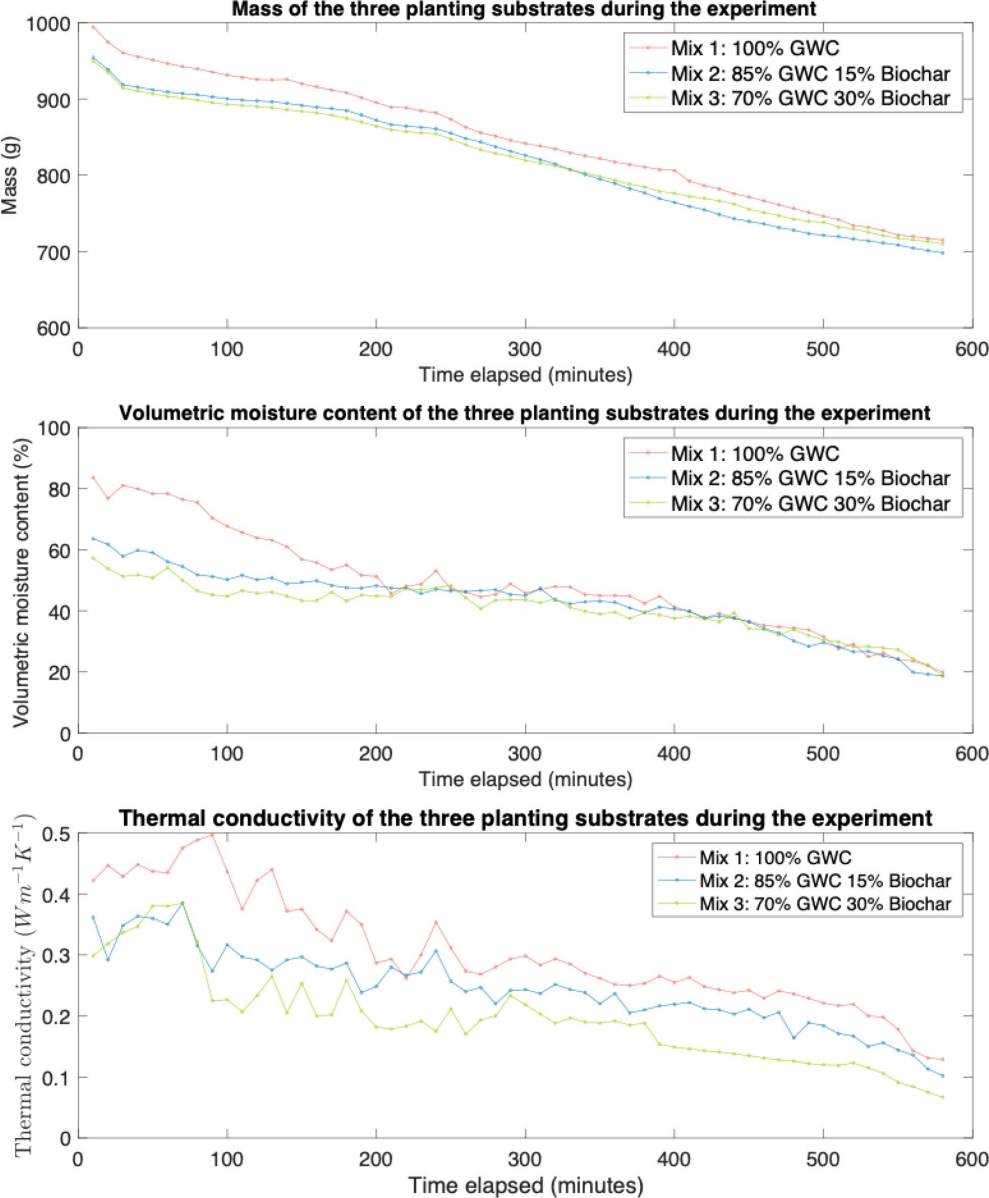
Mass, thermal conductivity and volumetric moisture content of the planting substrates during the experiment

The volumetric moisture content fell fastest for mix 1 but from a higher starting point (Fig. 3). Compared to their saturated state, mix 1 fell by 66.02% vol, mix 2 by 61.03% vol and mix 3 by 46.95% vol. From 220 min onwards, the results converged with the other mixes and volumetric moisture content remained similar for the remainder of the experiment. While measurements in a saturated state indicated that the addition of biochar lowers maximum saturation, it also has greater retention of the moisture.

The mass of the mixes steadily fell throughout the experiment, showing an almost linear relationship with time. Mix 1 began with the highest mass in its saturated state, falling by 31.54% after 580 min. Mixes 2 and 3 fell slightly less, at 28.21% and 28.13%, respectively. Because of the relationship between mass and gravimetric moisture content, this suggests mixes 2 and 3 saw proportionally smaller reductions in gravimetric moisture content, which matches the trend in volumetric moisture content. There was a positive, statistically significant (*p* ≤ 0.05) relationship between mass and volumetric moisture content for all three mixes.

As seen in Fig. 3, with the exception of one data point, mix 1 had the highest thermal conductivity among the three mixes throughout the drying procedure. Of the mixes that contained biochar, thermal conductivity was similar for the first 100 min, before the results diverged. Mix 3 had the lowest thermal conductivity, achieving 0.067 Wm^−1^ K^−1^ at the end of the experiment, compared to 0.102 Wm^−1^ K^−1^ for mix 2 and 0.129 Wm^−1^ K^−1^ for mix 1 with no biochar.

**Fig. 4:**
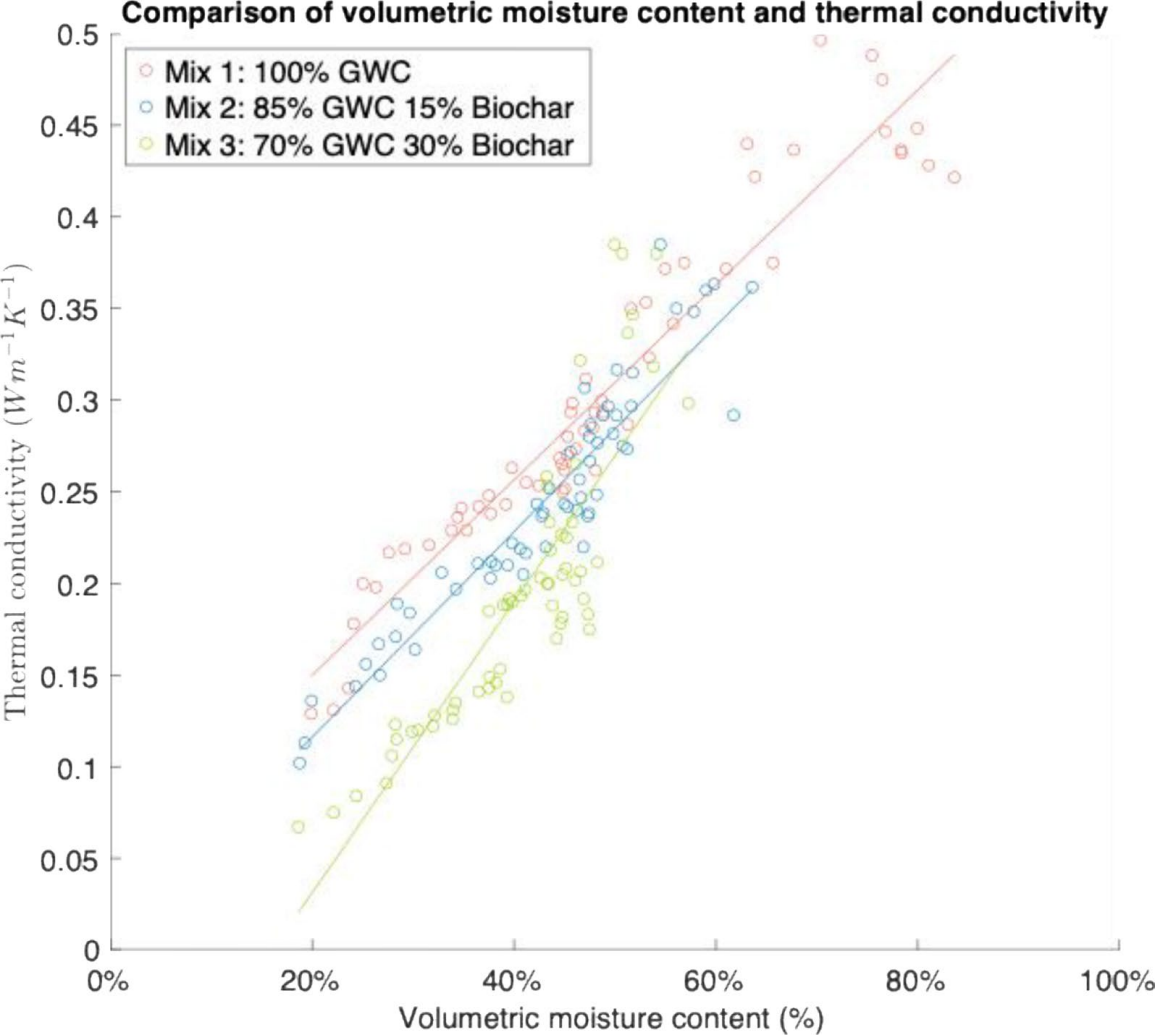
A scatter plot comparing volumetric moisture content and thermal conductivity

There was a positive, statistically significant (*p* ≤ 0.05) correlation between volumetric moisture content and thermal conductivity for all three mixes (Fig. 4). A linear model between volumetric moisture content and thermal conductivity was produced for each mix (Eq. 1). The linear model provided an excellent fit for mix 1 but weakened for mix 2 and mix 3, suggesting a non-linear relationship. Notably, mix 1 and 2 saw similar gradients of 0.0053 and 0.0047, respectively, while mix 3 with higher biochar content saw a steeper gradient of 0.0075. At high volumetric moisture levels, there was almost no differentiation in thermal conductivity, but when moisture content decreased, the thermal conductivity became far lower.

1 $$\begin{gathered} Mix{ }1:{ }y = 0.0457 + 0.0053x,R^{2} = 92.33{\text{\% , }} \hfill \\ Mix{ 2}:{ }y = 0.0405 + 0.0047x,{ }R^{2} = 74.44{\text{\% , }} \hfill \\ Mix{ 3}:{ }y = - 0.1103 + 0.0075x,{ }R^{2} = 73.26{\text{\% }} \hfill \\ \end{gathered}$$

Equation 1: Linear regression model results for each mix.

### Statistical modelling

A least-squares multivariate linear regression model predicts thermal conductivity from the independent variables of volumetric moisture content and biochar fraction. The model fit was good (R^2^ = 85.03%) and each independent variable had a statistically significant effect on thermal conductivity (Table 5). Accuracy was reasonable, with a Root Mean Squared Error (RMSE) of 0.0349 Wm^−1^ K^−1^. The biochar additions reduced thermal conductivity, while volumetric moisture content increased it.

**Table 5:** Regression results for multivariate linear regression model of thermal conductivity (*p ≤ 0.05)

	Estimated coefficient	Standard error	*t*-stat	*p*-value
Coefficient	0.0376*	0.0108	3.4756	0.0216
Biochar fraction (%)	−2.1100*	0.0224	−9.4344	0.0000
Volumetric moisture content (%vol)	0.0054*	0.0002	26.6257	0.0000

**Fig. 5:**
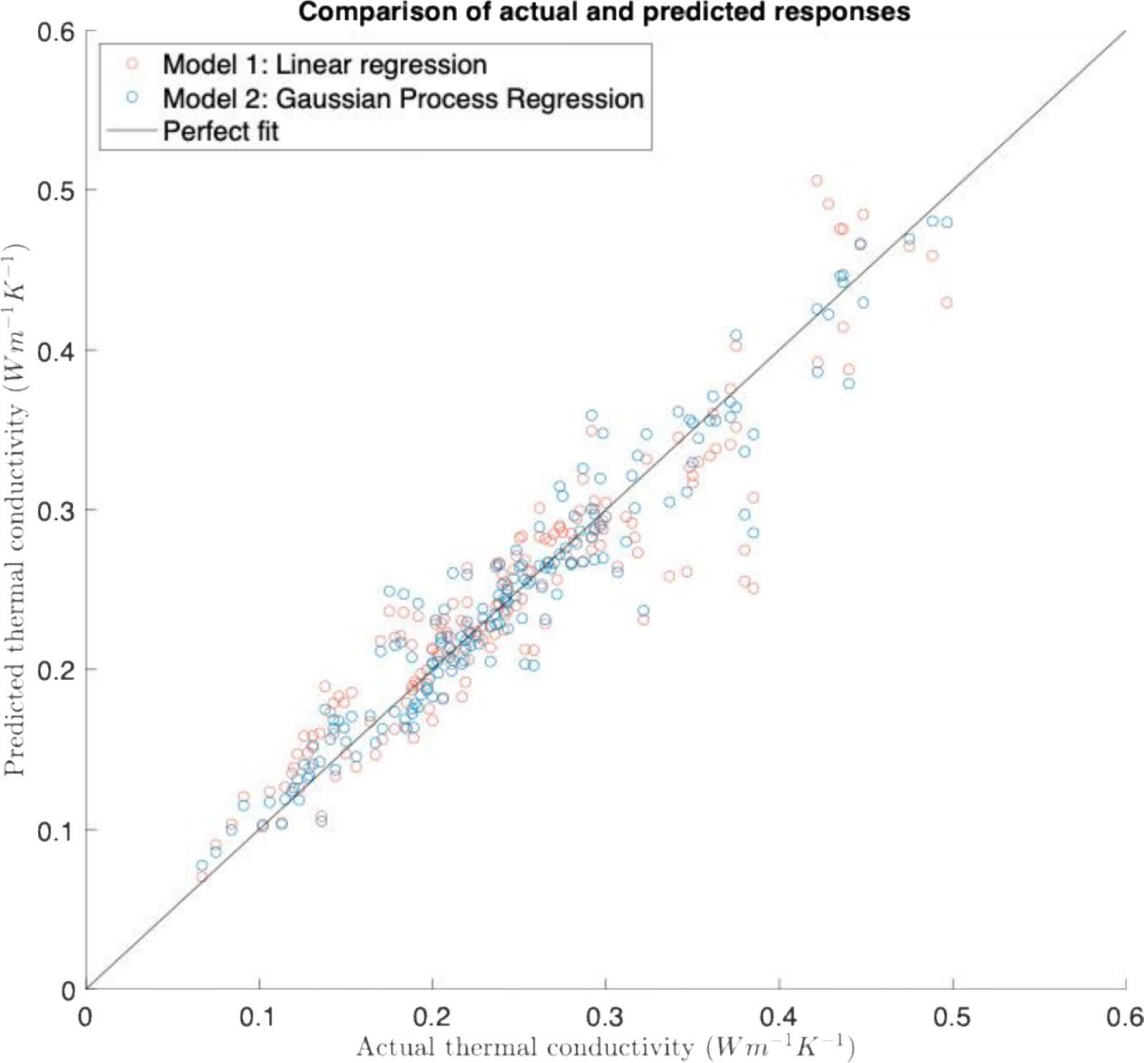
Plot of actual and predicted responses for the linear regression model and Gaussian Process Regression model

To understand whether a non-linear model could better explain the results, the model was compared with a Gaussian Process Regression model. This was associated with an improvement in RMSE to 0.0248 Wm^−1^ K^−1^, which indicates that the relationships between biochar fraction, volumetric moisture content and thermal conductivity are better explained by the non-linear model (Fig. 5).

### Application to a living wall system

2 $$R_{n} = \frac{d}{\lambda },{\kern 1pt} \;R_{total} = R_{si} + R_{1} + \ldots + R_{n} + R_{se}$$

Equation 2: Equation for the R-value of each homogenous component within a wall (${\varvec{\lambda}}$ thermal conductivity, d thickness, R_si_ internal surface resistance, R_se_ external surface resistance).

To understand the potential impact on the design of LWSs, the data are practically applied to calculate the overall resistance provided by the substrate (Anderson and Kosmina 2019). This treats the LWS as a construction layer, with a typical substrate thickness of 100 mm being assumed (Vijayalaxmi and Gandham 2023).

**Fig. 6:**
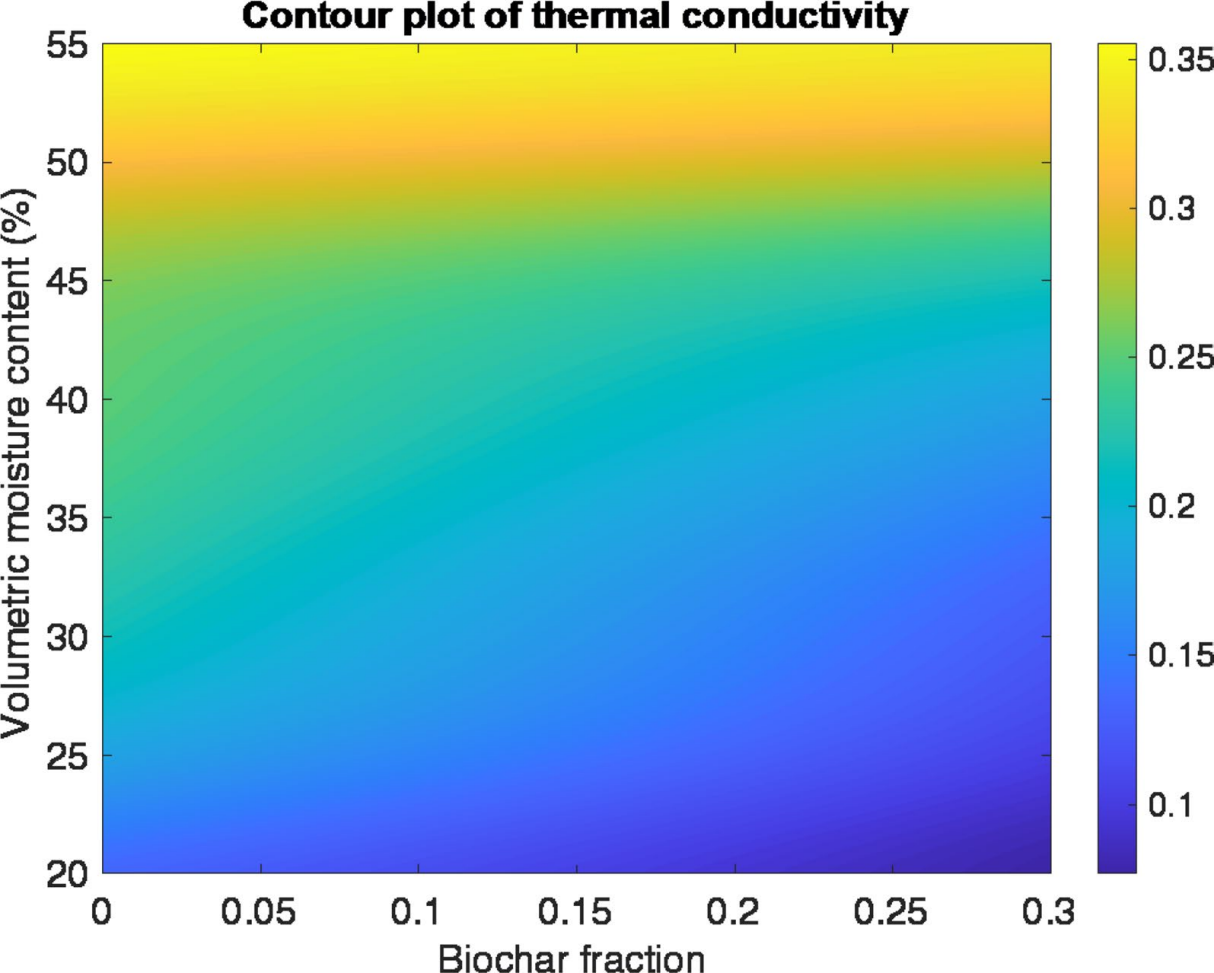
Contour plot of thermal conductivity of the planting substrate

**Fig. 7:**
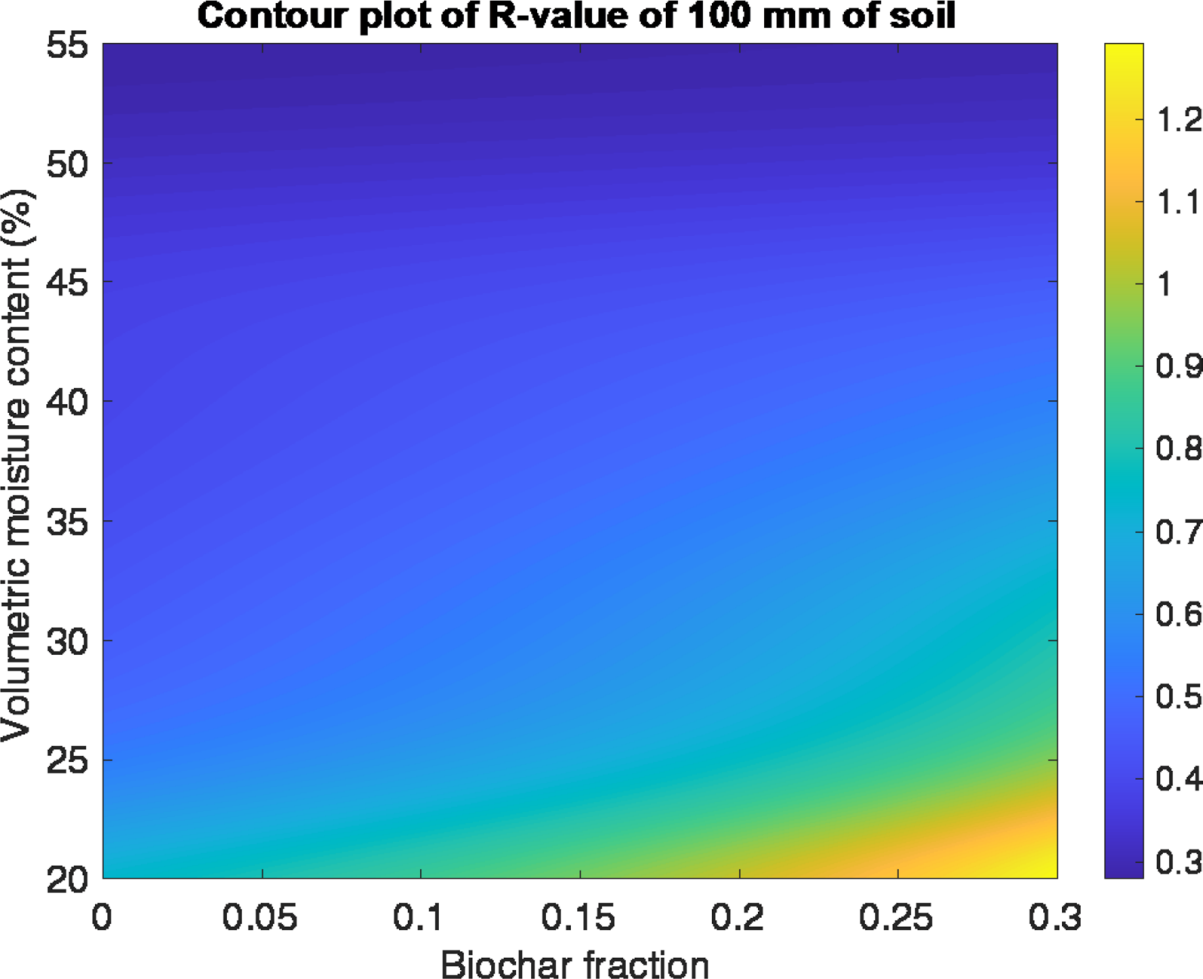
Contour plot of the R-value provided by 100 mm of planting substrate

The data suggest that increasing the biochar fraction and reducing moisture content in the substrate will minimise its thermal conductivity (Fig. 6). The model indicates the 30% biochar mix (mix 3) at 30% volumetric moisture content would achieve a thermal conductivity of 0.12 Wm^−1^ K^−1^, and when applied at 100 mm thickness with LWS would provide 0.82 m^2^ KW^−1^ of added thermal resistance to a wall (Fig. 7). At the same moisture level, GWC with no biochar (mix 1) would achieve 0.22 m^2^ KW^−1^. This indicates that the addition of 30% biochar to the planting substrate would almost halve the thermal conductivity. This data could be used as part of a U-value calculation of a whole wall construction; however, the overall U-value would depend on the thermal properties of the other construction layers and the ventilation of any air spaces within the wall construction.

## Discussion

The findings show a statistically significant difference in the conductivity between the three planting substrates depending on the biochar fraction and support earlier studies (Usowicz et al. 2016; Lunt et al. 2023), which highlighted biochar’s potential insulating properties. The biochar mixes tended to contain larger particle sizes than the GWC only mix, supporting previous research (Ng and Low 2010; Lunt et al. 2023) which found that larger particle sizes increased porosity. Increased porosity reduced thermal conductivity by acting as a barrier to convection currents. Added biochar was associated with greater reductions in thermal conductivity at lower moisture levels, with a relationship between biochar fraction, volumetric moisture content and thermal conductivity.

While only the mix of GWC tended to be more thermally conductive, it also held more water initially. The reduced moisture saturation of biochar mixes contrasts with previous research by Duang and Nguyen (2017) suggesting that biochar has greater ability to absorb and hold moisture. However, this may be explained by the higher proportion of larger particle sizes (4–16 mm) in the biochar substrates, which increased the textural coarseness of the substrate, thus lowering the moisture saturation potential of biochar substrates. Once fully saturated at the start of the experiment, the planting substrates with higher biochar content had a lower volumetric moisture content. The lowered saturation potential of biochar substrates translates to lower weight loads representing a significant advantage for the wider application of LWSs, consistent with the lower bulk density of biochar (Acharya et al. 2024). During the period 120–380 min, the moisture content of the biochar substrates did not decrease as steeply as that of the non biochar mix, with all mixes plateauing at around the same level (40–50%). The fact that higher moisture retention was associated with added biochar would significantly support LWSs, by reducing the demand for irrigation and maintenance requirements (Dede et al. 2021), and in moderating soil moisture at levels most suitable for plant performance. The non-linearity may be associated with the hydrophobicity of biochar at higher moisture levels (Adhikari et al. 2022), meaning that the addition of biochar increased water-holding capacity while moisture retention increased at lower levels due to capillary retention.

These findings suggest that biochar as a component of planting substrate may help negate the trade-offs between the moisture and thermal performance of LWSs by moderating the soil environment. Lower moisture might also benefit LWS plants by reducing the risk of waterlogging stress and plant disease (Murphy et al. 2024). However, improvements in substrate moisture moderation could result in a trade-off between insulation in winter and summer cooling: the lowered maximum moisture in planting substrates with higher biochar may provide additional thermal resistance, but it may also lower saturation, potentially reducing LWS transpiration and evaporative cooling from plants in the summer (Cameron et al. 2014). Lower evaporation could reduce the need for irrigation (Bustami et al. 2018), indirectly reducing the operational energy and carbon associated with water use in LWS (RICS 2023). This evidence builds on existing work suggesting that planting substrate cannot be considered on its own but alongside other choices in LWS design and must reflect site context (Dede et al. 2021; Jimenez et al. 2024).

At the end of the monitoring period, the mix with the highest addition of biochar (mix 3) achieved the lowest thermal conductivity of 0.067 Wm^−1^ K^−1^ with a mass of 698.3 g and a moisture content of 18.63% vol. This compares favourably with previous studies into the thermal conductivity of different LWSs substrates, which ranged from 0.051 Wm^−1^ K^−1^ for a specific LWS substrate made from coconut matting (Libessart and Kenai 2018) to 0.9 Wm^−1^ K^−1^ for wet, compacted GWC (Lunt et al. 2023).

Even after optimisation of the biochar fraction and moisture content, thermal conductivity was not comparable to modern insulation materials available for buildings. Expanded Polystyrene (EPS) and glass wool have thermal conductivities of 0.037 Wm^−1^ K^−1^ and 0.051 Wm^−1^ K^−1^ respectively (Yuan 2018), both of which are much lower than that of the substrate with 30% biochar at 30% volumetric moisture content’s thermal conductivity of 0.12 Wm^−1^ K^−1^. However, this may be acceptable given that LWSs support other benefits that are desirable to building owners and users (Manso et al. 2021). Where necessary to meet regulatory requirements such as Part L1 and Part L2 of the Approved Documents guidance in the UK for walls upgraded with external insulation (HM Government 2024), LWSs could also be combined with other insulation materials to reduce their thickness.

## Conclusions and perspectives

This paper assessed the influence of biochar addition on the thermal and moisture performance of three planting substrates, finding that additions of biochar cause non-linear reductions in thermal conductivity at lower moisture levels. Increased biochar could therefore serve as an extremely useful product for reducing the thermal transmittance of walls and the energy use of buildings fitted with LWSs. Biochar was shown to increase the moisture retention of planting substrate but was also associated with lower maximum saturation. Biochar could provide both direct energy performance benefits from added thermal resistance and indirect benefits from lower water requirements and improved plant performance. However, lower moisture content could be undesirable where LWSs are used for cooling due to lower transpiration and evaporative cooling, with trade-offs between reduced thermal conductivity and cooling efficiency.

The methodology provides clear evidence on the beneficial role of biochar for the thermal and hydrological characteristics of growth substrates for use in LWS. There are however areas which warrant further study, namely how the elevated emissivity of biochar may impact LWS evaporation, and how biochar additions to growing substrate might influence plant performance and the aerial and substrate thermal performance of LWS. Additionally, the long-term fate and thermal functionality of biochar in LWS requires further investigation given its promotion of microbial colonisation. This study focused on one type of biochar, further work should assess the thermal performance and LWS applicability of different types of biochar. Furthermore, future research could provide in situ data on the influence of biochar on LWS performance using a live case study and heat flux monitoring (Anderson and Kosmina 2019), to fully explore how biochar might be best used in the design, installation and maintenance of LWSs. This study also focussed on bare soil, with investigation needed on how biochar additions interact with different plant species given that biochar has been found to influence seed germination, plant growth and root development. Further work could also look beyond thermal and moisture performance, such as investigating and optimising the cost of biochar-enhanced LWSs.

The paper builds on previous work (Fox et al. 2022) to provide evidence on how the properties of different planting substrates used in LWSs affect thermal and moisture performance (Usowicz et al. 2016; Libessart and Kenai 2018; Lunt et al. 2023). The use of biochar in LWSs offers significant opportunity for improving thermal performance and lowering energy usage. The study focused on LWSs, the results are relevant to other forms of green infrastructure that include a planting substrate such as intensive green roofs.

## Data Availability

All data are currently available upon request and will be made available from the Plymouth Electronic Archive and Research Library (PEARL).

## Abbreviations

GWC: Green waste compost
LWS: Living wall system
PM: Particulate matter
VOC: Volatile organic compound
RMSE: Root mean squared error
